# Enhancement of root sulfur metabolic pathway by overexpression of *OAS-TL3* to increase total soybean seed protein content

**DOI:** 10.1007/s11032-022-01348-y

**Published:** 2023-01-12

**Authors:** Ye Zhang, Han-zhu Zhang, Abraham Lamboro, Jia-yu Fu, Ye-yao Du, Jing Qu, Pi-wu Wang, Yang Song

**Affiliations:** Joint Laboratory of International Cooperation in Modem Agricultural Technology of Ministry of Education, Plant Biotechnology Center, College of Agronomy, Jilin Agricultural University, Changchun, 130118 People’s Republic of China

**Keywords:** OAS-TL3, Sulfur metabolic pathway, Root system, Methionine, Protein content

## Abstract

Sulfur is essential for plant growth, and the uptake of sulfate by plant roots is the primary source of plant sulfur. Previous studies have shown that the OAS-TL gene is a key enzyme in the sulfur metabolic pathway and regulates cysteine (Cys) synthase production. However, the interaction mechanism of the glycine max *OAS-TL3* Cys synthase (*OAS-TL3*) gene on soybean root morphology construction and seed protein accumulation is unclear. This study shows that mutant M18 has better root growth and development, higher seed protein content, and higher methionine (Met) content in sulfur-containing amino acids than wild-type JN18. By transcriptome sequencing, the differentially expressed *OAS-TL3* gene was targeted in the mutant M18 root line. The relative expression of the *OAS-TL3* gene in roots, stems, and leaves during the seedling, flowering, and bulking stages of the *OAS-TL3* gene overexpression lines is higher than that of the recipient material. Compared to the recipient material JN74, the enzymatic activities, Cys, and GSH contents of OAS-TL are higher in the sulfur metabolic pathway of seedling roots. The receptor material JN74 is exogenously applied with different concentrations of reduced glutathione. The results demonstrate a positive correlation between reduced glutathione on total root length, projected area, surface area, root volume, total root tip number, total bifurcation number, and total crossing number. The Met and total protein contents of sulfur-containing amino acids in soybean seeds of the *OAS-TL3* gene overexpression lines are higher than those of the recipient material JN74, while the gene-edited lines show the opposite results. In conclusion, the *OAS-TL3* gene positively regulates soybean root growth, root activity, and the content of Met in the seeds through the OAS-TL-Cys-GSH pathway. It breaks the limitation of other amino acids and facilitates the increase of total seed protein content.

**Supplementary Information:**

The online version contains supplementary material available at 10.1007/s11032-022-01348-y.

## Introduction

Sulfur is an essential nutrient element for plant growth and development (Birke et al. 2012). Although the sulfur content only accounts for about 0.1% of plant dry weight, it significantly impacts the growth and development of plant cells, such as metabolism and synthesis (Chen et al. 1982). Metabolism of sulfur and carbon jointly coordinate to promote the balance of plant nutrients (Hawkesford and Kok 2006; Leustek et al. 2000). If the rice is deficient in sulfur, it can have slow greening speed, short plants, and late maturity, affecting the annual rice yield (Lunde et al. 2008). If maize is deficient in sulfur, yellow leaf clusters and young leaves appear, the plants are short, and maturity is delayed (Pagani and Echeverría 2012). Sulfur shortage causes the veins and leaves of the potato to turn yellow in a wide range, and the growth rate reduces. However, the leaves cannot fall off in advance, and brown spots may appear, similar to the symptoms of nitrogen deficiency (Pavlista 2005). In addition, sulfur deficiency can limit soybean leaf growth area, decrease chlorophyll content, decrease nitrate reductase activity, and block nitrogen metabolism, seriously affecting the growth and food quality of soybean (Li et al. 2004). Sulfur is an essential element in the active centers of many enzymatic reactions and an essential component of many plant compounds, cofactors, and secondary metabolites, such as iron and sulfur clusters, polysaccharides, sulfur lipids, vitamins, biotin, thiamine, S-adenosyl methionine (SAM), glutathione (GSH), chelating peptides, cysteine (Cys), and methionine (Met) (Kurmanbayeva et al. 2017; Kopriva 2006).

Sulfate in the soil is actively absorbed into plants through their huge root systems, and SO_4_^2−^ enters the sulfur metabolism process through a series of reduction and assimilation reactions of plants to generate Cys (Khare et al. 2017). Cys is a key sulfur-containing compound because it is both the endpoint of sulfur absorption and the starting point for synthesizing various sulfur-containing metabolites such as Met, GSH, and plant chelates (PCs) (Leustek et al. 2000; Howarth et al. 2003). The final reaction of the compound O-acetyl-L-serine (thiol)-lyase (OAS-TL) catalyzed the formation of Cys from hydrogen sulfide (H_2_S) and OAS (Bogdanove and Hell 1997; Droux et al. 1998). Current studies have shown that the *OASTL* gene participates in plant growth (García et al. 2010; Takahashi et al. 2011), photosynthesis (Hawkesford and Kok 2006), fertilization and disease resistance (Droux et al. 1998), oxidative stress, and other abiotic stresses (Droux 2004; García et al. 2010; Kim and Steudle 2007). For example, overexpression of the wheat *OASTL* gene can improve the ability of tobacco to adapt to oxidative stress (Youssefian et al. 2001). In Arabidopsis *OAS-A1* mutants, the decrease of intracellular L-Cys concentration changes the redox status of glutathione, resulting in the homeostasis of hydrogen peroxide in vivo (García et al. 2010). In addition, in the case of sulfur or nitrogen starvation stress (Droux et al. 1998; Takahashi et al. 2011; Dominguez-Solís et al. 2001), oxidative stress (Youssefian et al. 2001), and heavy metal stress, *OASTL* was found to play an important role in the process (Tian et al. 2016; Bennetzen et al. 2012). Overexpression of the *OASTL* gene may also lead to elevated thiosulfhydryl groups in several plant inverters (Dominguez-Solís et al. 2001).

Soybean provides about 30% of the vegetable protein for humans. Maintaining or improving the protein content of soybean is the prerequisite and primary goal of soybean protein improvement breeding (Zhang et al. 2018; Lee et al. 2019). The change from sulfur balance to sulfur deficiency is now common in soils worldwide (Scherer 2009). As a sulfur-loving crop, soybean is dependent on sulfur for nodulation, nitrogen fixation, and protein synthesis. Therefore, increasing the relative expression of the *OAS-TL3* gene may be important for cultivating new high-protein soybean germplasm. This study revealed that the *OAS-TL3* gene positively regulates the growth and development of the soybean root system and the sulfur-containing amino acid Met content through the OAS-TL-Cys-GSH pathway. The limitation of other amino acid content is broken, ultimately making a positive contribution to increasing the total content of grain protein.

## Materials and methods

### Plant materials and growing conditions

After extreme drought treatment of JN18 population, one plant with normal growth was found. To purify the genetic background, the M18 mutants were selfinged for four generations in an experimental field in Changchun, Jilin Province, China (43° 88ʹN, 125° 35ʹE). The sowing distance was 15 cm, with a row spacing of 4 m and a plant spacing of 3 rows. The experiment was replicated three times for each group. After 128 days of growth, the protein content and amino acid composition of JN18 and mutant material M18 were determined.

In the Plant Biotechnology Central Laboratory of Jilin Agricultural University, test materials were strictly selected. Furthermore, neat, healthy, and full T_2_ *OAS-TL3* overexpression materials OEA1 and OEA2 were selected. The vector materials KO1 and KO2 were edited. Seeds of recipient material JN74 were provided by the Plant Biotechnology Center of Jilin Agricultural University. The selected seeds were sown in a plastic bucket that was 15-cm high, 15 cm in upper diameter, and 10 cm in bottom diameter. Each barrel was filled with 1 kg of soil. There were 15 pots, with three plants left in each pot. The experiment was conducted in a completely randomized design at 25 ℃, 16 h of light, and 8 h of darkness. The indicators were measured after 4 weeks. After 130 days, the quality of each soybean strain was determined.

The 60 WT (JN74) seeds were put into 3 culture dishes (9 cm × 1.5 cm) on average and incubated in dark at 25 ℃ for 4 days. Selected the seeds with the same germination condition and put them into different concentrations of reduced GSH (0 ug/mL, 2.5 ug/mL, 5.0 ug/mL, and 10 ug/mL) for water culture. Each concentration is 10 seedlings. The seeds were exposed to light at 25 ℃ for 16 h and dark for 8 h. After 9 days, each root index is determined.

### RNA extraction and quantitative real-time PCR (qRT-PCR) analysis

The total RNA was extracted and reverse transcribed according to Nie et al. (2019), and qRT-PCR was conducted using BioEasy Master Mix (Bioer Technology) in a CFX96 real-time system (Bio-Rad) following the manufacturer’s instructions. Each treatment comprised three biological and three replicate samples. We used the Actin2 gene as an internal control. The specific primer sequences are F: CTGAGGTTCTATTCCAGCCATCC, R: CCACCACTGAGGACAACATTACC. In addition, the specific primer sequences of the target gene *OAS-TL3* are as follows: F: AGGCACAGTCTCTGGGGTTG, R: CAGGTTTACCATTCAGCAC. Relative expression levels were calculated using 2^−ΔΔCt^ method (Zhang et al. 2022).

### Phylogenetic analysis

The BLAST homologous sequence alignment obtained from transcriptome sequencing by NCBI (https://www.ncbi.nlm.nih.gov/) revealed highly homologous with the *OAS-TL3* gene, proposed as *OAS-TL3* (Gene ID:100,101,899). Swiss model software was used to predict the tertiary structure of *OAS-TL3* encoded protein. The phylogenetic tree was constructed by the neighbor-joining (NJ) method using MEGA7.0 software (http://www.megasoftware.net/download _form).

### Expression vector construction and plant transformation

First, the pMD-18 T-OAS-TL3 cloning vector was constructed with the specific primers of F: TAATGGCTTCCCTCA and R: TTAATCAACTGCTACTGGC. Then, the pCAMBIA3301-OAS-TL3 overexpression vector was constructed with the specific primers: F: agaacacgggggactcttga CCATGGTAATGGCTTCCCTCA and R: cgatcggggaaattcgagctGGTCACCTTAATCAACTGCT ACTGGCPCR. The constructed overexpression vector was verified by colony PCR (Fig. S1) and plasmid through double digestion (Fig. S2). Finally, the successful pCAMBIA3301-OAS-TL3 (Fig. S3) recombinant vector was detected. The gene-editing vector pCBSG015-OAS-TL3 (Fig. S5) (Numi Biotechnology Co., LTD.) was established, and the target verification of the editing vector (Fig. S4) (Jilin Kumei Technology Co., LTD.) was performed. The constructed vector was transferred into Agrobacterium EHA105 to transform cotyledon explants of “JN74” using the method described by Zhao et al. (2016) and Dai et al. (2018).

### Determination of OAS-TL activity, Cys, and GSH content

Seed root samples (approximately 0.5 g) were collected in a 5 mL centrifuge tube in liquid nitrogen. The OAS-TL (EC number:2.5.1.47) enzyme activity was measured using the method described by Marrero-Degro et al. (2011), Cys content was measured according to Gaitonde (1967), and GSH content was measured according to Anderson et al. (1976).

### Determination of root phenotype and activity at seedling stage and dry weight of above-ground part of the soybean

A digital scanner was used to scan the root system of different transgenic lines, and root length, diameter, surface area, volume, surface area, total number of root tips (TNT), total number of root forks (TNF), and total number of root crossings (TNC) were analyzed using the DJ-GXG02 root image analysis system. After scanning, the plants were placed in the oven, dried to a constant weight at 75 ℃, and the dry weight of the above-ground part was measured. TTC (2, 3, 5-tripheyl tetrazolium chloride) method was used to measure the root activity of soybean seedlings of different strains. The determination methods were based on the study of Knievel (1973).

### Determine protein content and amino acid composition

The protein content and amino acid components of soybean seeds (such as Asp, Ala, Arg, Cys, Glu, Gly, His, Ile, Leu, Lys, Met, Phe, Pro, Ser, Taa, Thr, Try, Tyr, and Val) were measured by FOSS near-infrared analyzer (Denmark FOSS Group). The specific determination method was referred to Xi et al. (2009).

### Statistical analysis

Data from this study were analyzed using the SPSS version 17.0 and the Student’s *t*-test. The mean values and standard errors were calculated, and *P* < 0.05 was considered statistically significant compared with WT plants.

## Results

### Phenotypic identification of soybean JN18 and mutant M18

**Fig. 1:**
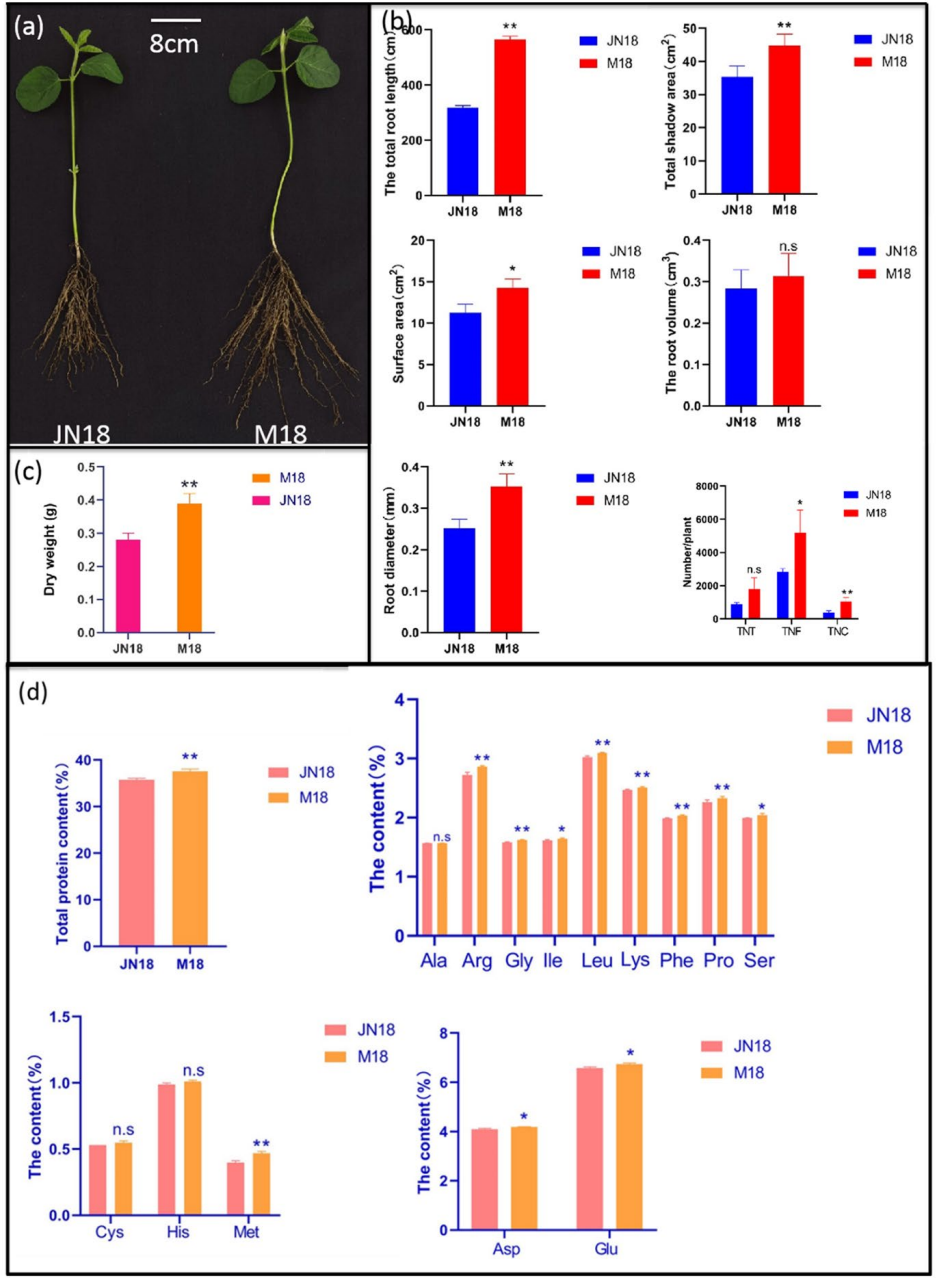
Phenotypic characteristics and protein content and amino acid groups in seeds of soybean JN18 and M18 at seedling stage. **a** Phenotypic characterization of wild-type and the M18 plants. Scale = 8 cm. **b** Statistical analysis of total root length, surface area, projected area, volume, diameter, total root tip number, crossing number, and bifurcation number of JN18 and M18 underground parts. **c** Statistical analysis of dry weight of JN18 and M18 overground parts. **d** Statistical analysis of protein content and amino acid components in JN18 and M18 grains. Error bar represents ± SD, *n* = 3, n.s indicates no significant difference, *P < 0.05, **P < 0.01 (Student’s *t*-test)

Due to the excessive drought in the experimental field, large plants in the JN 18 pilot area were dead, but one plant had rich green leaves and was growing normally. These traits were found to be stable after 4 generations (Fig. 1a), and the phenotypes of the mutant plant were determined. The results show that the total root length, surface area, total projected area, diameter, total transverse root number, and total root tip number of mutant M18 were significantly higher than those of wild-type soybean JN18 at the seedling stage (Fig. 1b). The dry weight of the above-ground part of mutant M18 was also significantly higher than that of wild-type soybean JN18 (Fig. 1c). Compared to wild-type soybean JN18, the protein content and Arg, Gly, Leu, Lys, Phe, Pro, and Met were significantly higher in the T_4_ mutant. Ile, Ser, Asp, and Glu were also significantly higher (Fig. 1d).

### OAS-TL3 Cys synthase gene

**Fig. 2:**
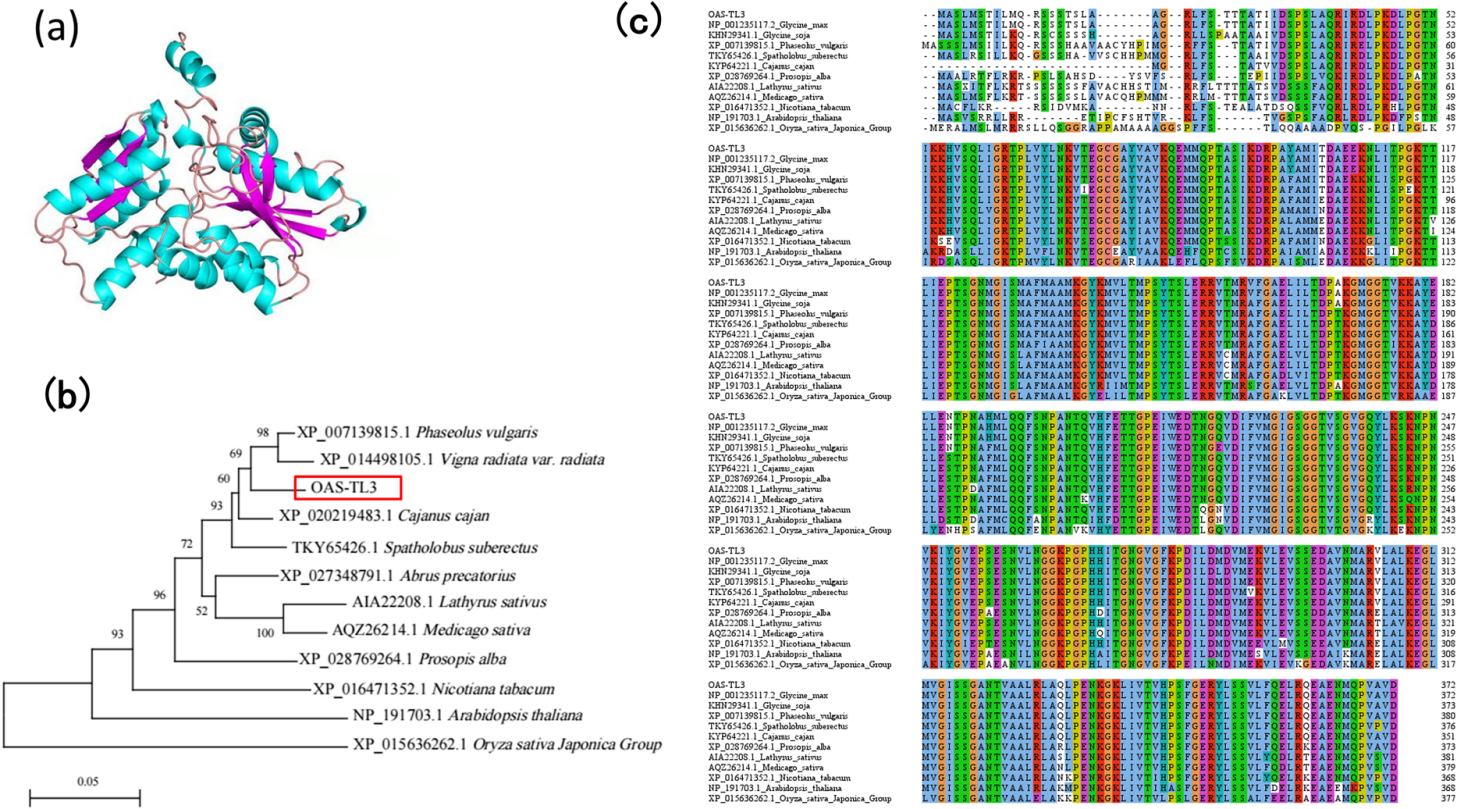
*OAS-TL3* genes analysis. **a** Tertiary structure prediction of *OAS-TL3* gene protein. **b** Phylogenetic tree of *OAS-TL3*in different species (plotting scale: 0.05). **c** Comparison of the similarity of *OAS-TL3* protein sequences in different species by DNAMAN software. The following species are included in the analysis (the GenBank accession numbers are shown in parentheses): *Glycine max* (NP_001235117.2), *Glycine soja* (KHN29341.1), *Phaseolus vulgaris* (XP_007139815.1), *Spatholobus suberectus* (TKY65426.1), *Lathyrus sativus* (AIA22208.1), *Medicago sativa* (AQZ26214.1), *Prosopis alba* (XP_028769264.1), *Cajanus cajan* (KYP64221.1), *Nicotiana tabacum* (XP_016471352.1), *Arabidopsis thaliana* (NP_191703.1), and *Oryza sativa Japonica Group* (XP_015636262.1)

By transcriptome sequencing, the *OAS-TL3* gene, which was mapped to be differentially expressed in the roots of the mutant material M18, had a total length of 1432 bp and an ORF length of 1119 bp, encoding 372 amino acids. The sequencing data is available in the NCBI database (http://www.ncbi.nlm.nih.gov/bioproject/903970). Number (PRJNA903970). The protein was 38.44% α helix (Hh), 17.20% extended chain (Ee), 35.22% random curl (Cc), and 9.14% β rotation (Tt). The protein encoded by the *OAS-TL3* gene within the genera has a stable spatial conformation based on the α-helix structure (Fig. 2a). The results showed that *OAS-TL3* had the closest genetic relationship with *Cajanus cajan*, with the highest homology of 96.02%. In addition, the homology between *OAS-TL3* and *Phaseolus vulgaris*, *Spatholobus suberectus*, and *Lathyrus sativus* was by 93.12%, 91.53%, and 91.45%, respectively (Fig. 2c). In order to understand the phylogenetic relationship between soybean *OAS-TL3* and homologous proteins of other species, a phylogenetic tree was constructed based on the amino acid sequence of *OAS-TL3*. The results showed that *OAS-TL3* belonged to the same branch as other legumes and had the closest genetic relationship with wild soybean (Fig. 2b).

### Detection of transgenic lines and quantitative analysis of OAS-TL3 gene

*OAS-TL3* plants of the T_2_ generation were tested for *Bar* gene, promoter *35 s*, terminator *NOs*, and Southern blotting. The sequences of the specific primers are shown in the attached table (Table S1). As can be seen from the Southern blotting results in Fig. S6, plants in lanes 1 and 4 were integrated into a single copy, and the named expression lines were OEA1 and OEA2. Target detection of transgenic *OAS-TL3* gene-edited plants showed that target 2 of KO1 edited line had 'A' base inserted, target 1 of KO2 edited line had “T” base deleted, and target 2 underwent “G-C” base replacement (Fig. S7) (Jilin Kumei Technology Co., LTD).

**Fig. 3:**
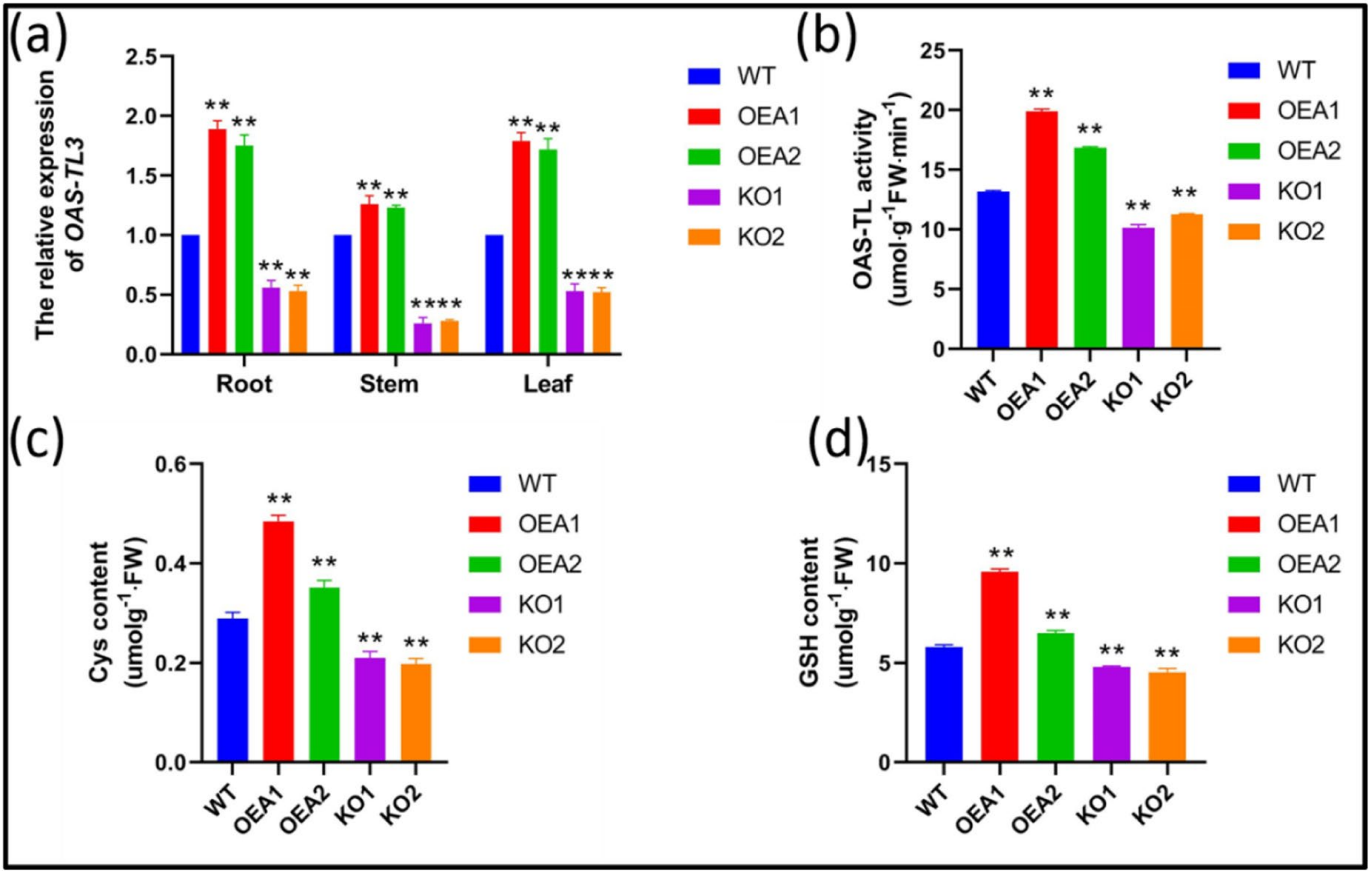
Related substances in sulfur assimilation pathway and *OAS-TL3* genes expression analysis of WT and transgenic line. **a** Expression level of *OAS-TL3* genes in WT and transgenic line. **b** Activity of OAS-TL enzyme in WT and transgenic lines. **c** Content of Cys in WT and transgenic lines. **d** Content of GSH in WT and transgenic lines. Error bar represents ± SD, *n* = 3, n.s indicates no significant difference, *P < 0.05, **P < 0.01 (Student’s *t*-test)

The detected transgenic plants were tested by qRT-PCR. The results showed that the relative expression of *OAS-TL3* increased by 89% and 75% in OEA1 and OEA2 soybean roots, respectively, by 26% and 23% in stems, and by 79% and 72% in leaves. The relative expression of *OAS-TL3* genes decreased by 44% and 47% in KO1 and KO2 soybean roots, respectively, by 74% and 72% in stems, and by 47% and 48% in leaves (Fig. 3a). In conclusion, the expression level of the *OAS-TL3* gene was increased in the overexpression lines, while the target location of the *OAS-TL3* gene was lost in gene editing lines, but the *OAS-TL3* gene was still partially expressed.

### Positive regulation of OAS-TL activity and Cys and GSH content in soybean roots by OAS-TL3

OAS-TL enzyme activity detection in soybean roots with different *OAS-TL3* transfers was conducted. The results showed that compared with control material WT, the OAS-TL enzyme activity in soybean roots with overexpressed lines OEA1 and OEA2 increased by 51% and 28%, respectively. The activity of the OAS-TL enzyme in the root system of gene-edited lines KO1 and KO2 at the seedling stage decreased by 23% and 14%, respectively (Fig. 3b).

The content of Cys in the root system of the *OAS-TL3* soybean was determined. The results showed that the content of Cys in the root system of OEA1 and OEA2 overexpressed soybean lines was 68% and 21%, respectively, which was higher than that of the control material WT. The gene-edited soybean lines KO1 and KO2 at the seedling stage showed lower Cys content in the seedling roots than the control group, decreasing by 27% and 31%, respectively (Fig. 3c).

Compared with the control group, the GSH content of *OAS-TL3* soybean root at the seedling stage was increased by 65% and 12%, respectively. The content of GSH in the root system of gene-edited lines KO1 and KO2 at the seedling stage decreased by 17% and 22%, respectively (Fig. 3d).

In conclusion, these results indicate that the *OAS-TL3* gene has a positive regulatory effect on the OAS-TL-Cys-GSH pathway in soybean seedling roots.

### Promotion of root growth and above-ground dry matter accumulation by the OAS-TL3 gene in soybean roots

**Fig. 4:**
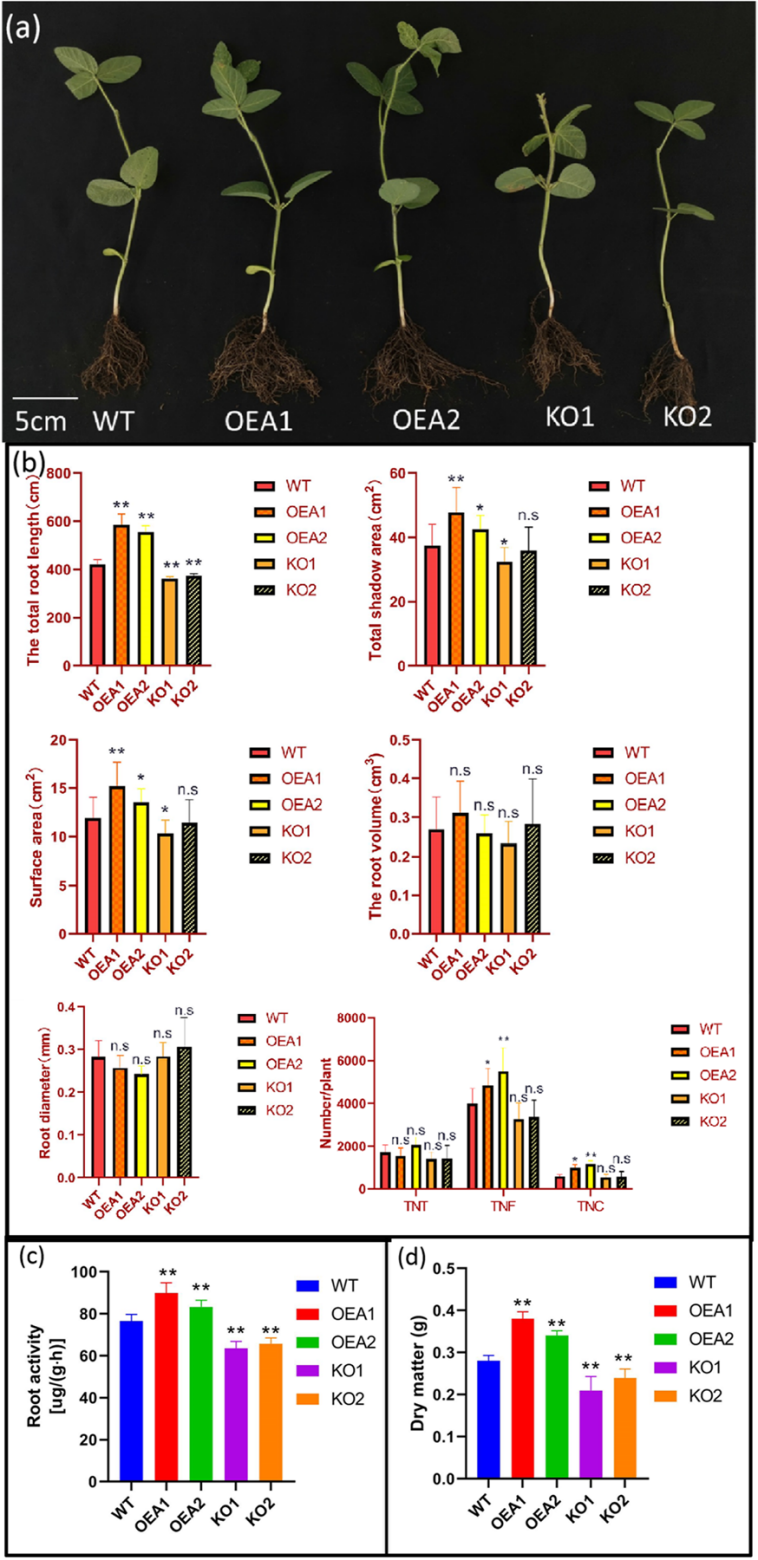
Above-ground plant and root analysis of WT and transgenic line. **a** Phenotypic analysis of plant WT and transgenic lines. Scale = 5 cm.** b** Total root length, surface area, projected area, volume, diameter, total root tip number, crossover number, bifurcation number of WT, and transgenic line. **c** Root activity analysis of WT and transgenic lines. **d** Dry matter quantity analysis of WT and transgenic lines. Error bar represents ± SD, *n* = 3, n.s indicates no significant difference, *P < 0.05, **P < 0.01 (Student’s *t*-test)

To explore the effect of the *OAS-TL3* gene on plant phenotype, we measured the morphological formation of soybean root and dry matter accumulation of the above-ground part. The results showed that the overexpressed lines OEA1 and OEA2 were higher than the control material WT in terms of total root length, projected area, surface area, the total number of forks, and the total number of crosses (Fig. 4b). Conversely, the edited expressed lines KO1 and KO2 were lower than the control material in total root length, projected area, and surface area (Fig. 4b). Compared with the control material WT, the T_2_ overexpressed lines OEA1 and OEA2 had more robust and developed roots in the underground part, and the above-ground part had thick stems and dark green leaves (Fig. 4a). However, the edited expression lines KO1 and KO2 showed relatively poor root growth and relatively short above-ground stems (Fig. 4a).

Analysis of root activity of different transgenic *OAS-TL3* lines showed that compared with the control material, the root activity of overexpressed lines OEA1 and OEA2 increased by 18% and 9%, respectively, while the root activity of edited expression lines KO1 and KO2 decreased by 17% and 14%, respectively (Fig. 4c). Compared with the control material WT, the dry matter accumulation of overexpressed above-ground lines OEA1 and OEA2 increased by 36% and 21%, respectively, while the edited expression lines KO1 and KO2 decreased by 25% and 14%, respectively (Fig. 4d). In conclusion, the increase of *OAS-TL3* gene expression may promote the morphological establishment of soybean roots. The primary root growth and lateral root elongation are enhanced, and the root activity is improved, facilitating nutrient absorption and dry matter accumulation of above-ground parts.

### Promotion of soybean root growth by exogenous application of GSH

**Fig. 5:**
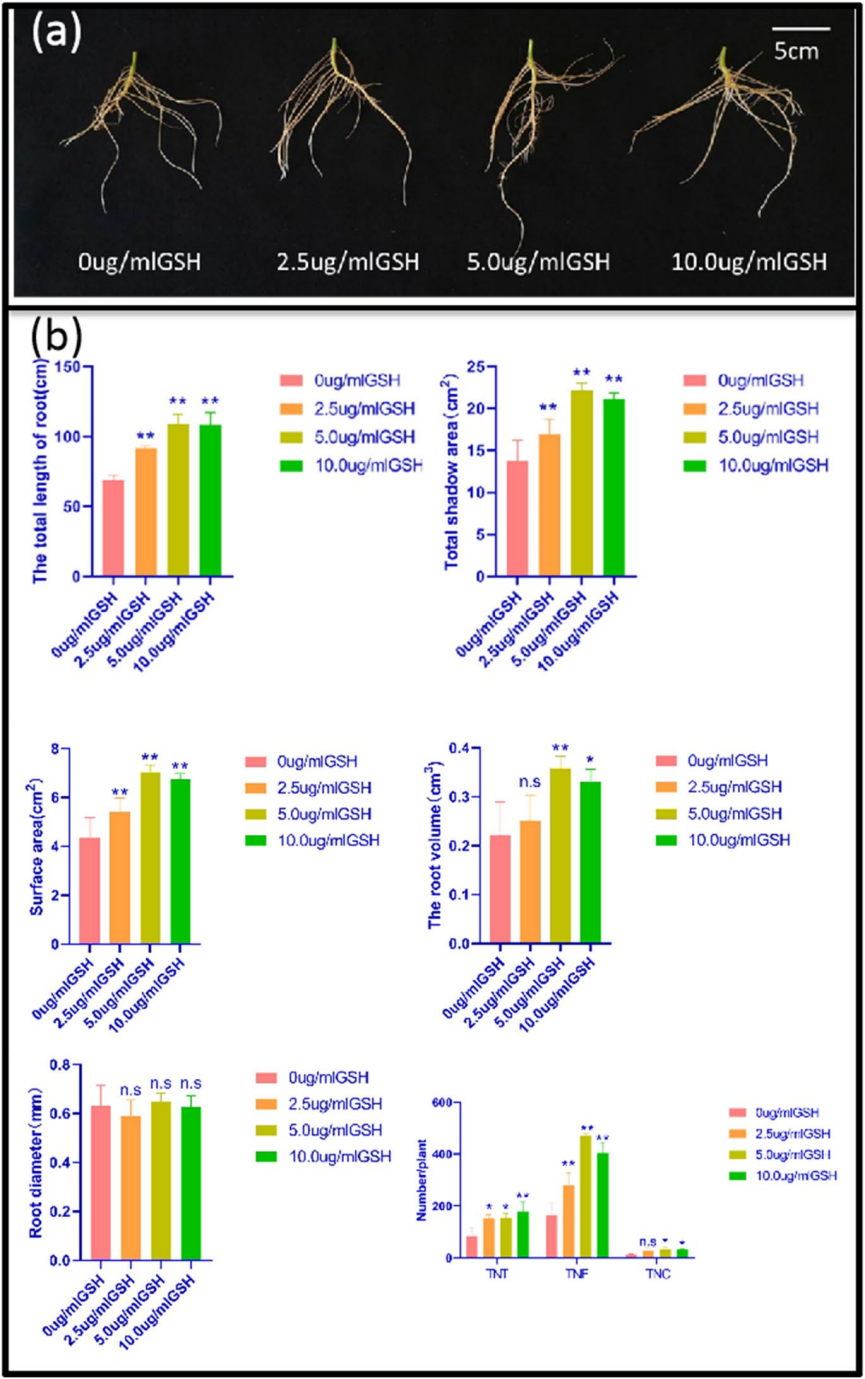
Phenotypic analysis of roots under WT (0 ug/mL) and different concentrations of GSH (2.5 ug/mL, 5.0 ug/mL, 10 ug/mL) stress. **a** Phenotypic analysis of roots under WT (0 ug/mL) and different concentrations of GSH (2.5u g/mL, 5.0 ug/mL, 10 ug/mL) stress. Scale = 5 cm. **b** Total root length, surface area, projected area, volume, diameter, total root tip number, crossover number, bifurcation number of WT (0ug/mL), and different concentrations of GSH (2.5ug/mL, 5.0ug/mL, 10 ug/mL) stress. Error bar represents ± SD, *n* = 3, n.s indicates no significant difference, *P < 0.05, **P < 0.01 (Student’s *t*-test)

Different concentrations of GSH (0 ug/mL, 2.5 ug/mL, 5.0 ug/mL, 10 ug/mL) were applied externally to the untransformed recipient material JN74. Compared to the soybean roots treated with GSH at 0 ug/mL, those treated with 2.5 ug/mL, 5.0 ug/mL, and 10 ug/mL concentrations of GSH showed significant differences in total root length, projected area, surface area, root volume, total root tip number, total bifurcation number, and total crossing number (Fig. 5a). The total root length, projected area, surface area, total root tip number, and total bifurcation number of soybean roots treated with 2.5 ug/mL GSH increased by 33%, 23%, 23%, 83%, and 69%, respectively. The total root length, projected area, surface area, volume, total root tip number, total fork number, and total cross number of soybean roots treated with 5.0 ug/mL GSH increased by 59%, 69%, 61%, 64%, 84%, 184%, and 210%, respectively. The total root length, projected area, surface area, volume, total root tip number, total bifurcation number, and total crossing number of soybean roots treated with 10 ug/mL GSH increased by 57%, 54%, 54%, 50%, 112%, 143%, and 213%, respectively (Fig. 5b). In conclusion, exogenous application of a certain concentration of reducing GSH can promote the elongation of taproots and the growth of lateral roots, which is conducive to the morphological formation of roots.

### Promotion of the total grain protein content in soybean roots by OAS-TL3 gene

**Fig. 6:**
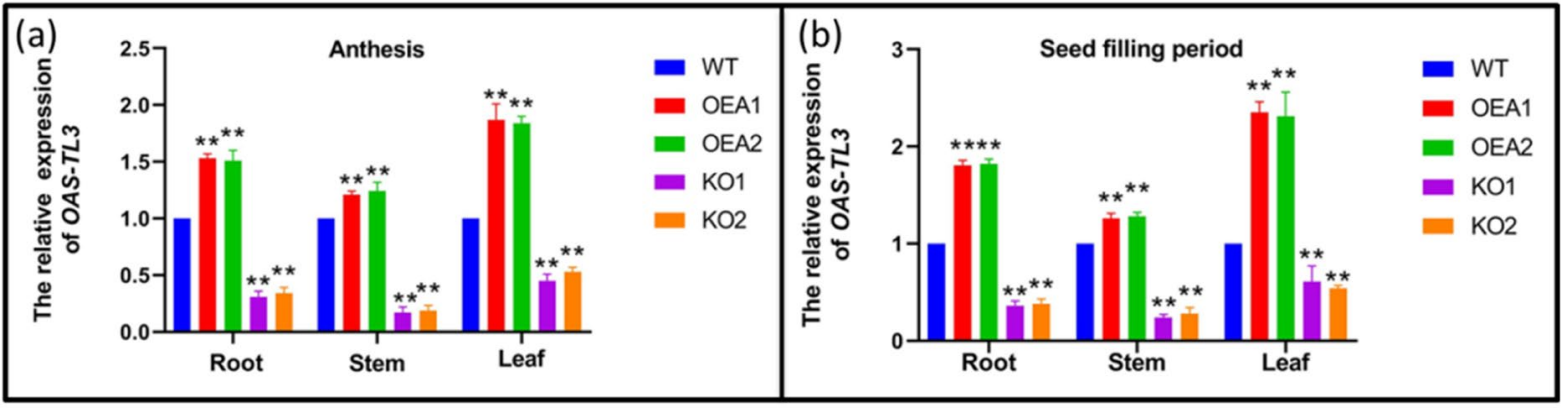
Expression analysis of *OAS-TL3* gene in WT and transgenic lines. **a** Analysis of *OAS-TL3* gene expression in WT and transgenic lines at anthesis stage. **b** Analysis of *OAS-TL3* gene expression in WT and transgenic lines at seed filling period. Error bar represents ± SD, *n* = 3, n.s indicates no significant difference, *P < 0.05, **P < 0.01 (Student’s *t*-test)

The expression level of the *OAS-TL3* gene in the transgenic lines OEA1 and OEA2 was measured at the flowering and grain bulking stages. The results showed that the expression levels of the *OAS-TL3* gene were higher in the roots, stems, and leaves of the overexpressed lines OEA1 and OEA2 than in the control WT material. The expression levels of *OAS-TL3* in roots, stems, and leaves of gene-edited lines KO1 and KO2 were lower than those of WT materials in the control group (Fig. 6a, b). Compared to the control material WT, the total protein content of OEA1 and OEA2 in overexpressed lines increased by 8.60% and 8.03%, while the protein content of KO1 and KO2 in gene-edited lines decreased by 6.93% and 5.74%. Compared with control material WT, there was no difference in the content of Cys in sulfur-containing amino acids of OEA1 and OEA2, but the content of Met was increased. Moreover, the contents of Ala, Asp, Leu, Phe, Glu, and Val in other amino acid components were increased. There was no difference between Cys and Met in sulfur-containing amino acids of gene-edited lines KO1 and KO2, while the contents of Ala, Asp, Leu, Phe, Glu, Val, and Pro were decreased (Table 1). In conclusion, the increased gene expression of *OAS-TL3* facilitates the accumulation of sulfur amino acid content and total protein content in soybean grain.

**Table 1:** Analysis of total protein content and amino acid components in soybean grains among different transgenic lines

	WT	0EA1	OEA2	KO1	KO2
Protein (%)	40.1 ± 0.26	43.55 ± 0.27**	43.32 ± 0.27**	37.32 ± 0.29**	37.80 ± 0.09**
Cys (%)	0.54 ± 0.12	0.55 ± 0.00	0.55 ± 0.00	0.53 ± 0.00	0.53 ± 0.00
Met (%)	0.44 ± 0.01	0.52 ± 0.02*	0.53 ± 0.00*	0.44 ± 0.00	0.46 ± 0.01
Ala (%)	1.68 ± 0.03	1.78 ± 0.01**	1.75 ± 0.01**	1.63 ± 0.01*	1.65 ± 0.01*
Asp (%)	4.55 ± 0.06	4.86 ± 0.03*	4.81 ± 0.02*	4.31 ± 0.03*	4.34 ± 0.00*
Leu (%)	3.26 ± 0.03	3.49 ± 0.01*	3.45 ± 0.01*	3.13 ± 0.02*	3.15 ± 0.00*
Phe (%)	2.14 ± 0.01	2.3 ± 0.02*	2.28 ± 0.01*	2.07 ± 0.01*	2.08 ± 0.00*
Glu (%)	7.49 ± 0.07	8.1 ± 0.05*	8.05 ± 0.02*	7.10 ± 0.03*	7.12 ± 0.00*
Val (%)	1.87 ± 0.01	1.98 ± 0.01*	1.97 ± 0.01*	1.81 ± 0.01*	1.83 ± 0.00*
Pro (%)	2.22 ± 0.06	2.29 ± 0.01	2.26 ± 0.01	2.08 ± 0.02*	2.11 ± 0.00*

Analysis of differences between transgenic lines and recipient material WT, mean value ± SD, *n* = 3, *P < 0.05, **P < 0.01 (Student’s *t*-test)

## Discussion

Soybean is the source of 70% of the world’s total protein diet. It meets or exceeds the criteria for all amino acids except for the first limiting amino acid Met and the second limiting amino acid tryptophan. Protein quality depends on the balance between essential amino acids. As the first limiting amino acid, Met limits and reduces the availability of other amino acids. Cys is another sulfur-containing amino acid in the protein. Although it is not an essential amino acid, it can save and replace the utilization of Met. The main function of protein in nutrition is to provide sufficient amino acids (Frideman and Brandon 2001). Improving the nutritional quality of soybean seed storage protein is to improve the relative content of sulfur-containing amino acids to make them balanced. It is of great significance in improving the nutritional quality and protein utilization rate of soybean.

In this study, protein and amino acid components were measured in grains of mutant M18 (Fig. 1a) with a developed JN18 root system. The results showed that Met content in soybean seeds of mutant M18 increased by 17.45%, higher than that of wild-type material, while Cys content was not different from that of wild-type material. Protein and Arg, Gly, Leu, Lys, Phe, and Pro were higher than those of wild-type materials (Fig. 1d). Because higher plants mainly absorb sulfates from the soil and synthesize sulfur-containing compounds through roots. We cloned the differentially expressed *OAS-TL3* gene in the root of mutant material M18. The ORF of this gene is 1119 bp long and encodes 372 amino acids. The *OAS-TL3* gene was considered a candidate gene for controlling the development phenotype of the M18 root system and increasing grain protein content. In order to further verify the function of the *OAS-TL3* gene, the *OAS-TL3* overexpressed lines OEA1 and OEA2 were obtained using Agrobacterium tumefaciens-mediated “JN74” soybean cotyledon explants and replicated in single copy form (Fig. S6). The gene-edited line KO1 was inserted with the “A” base at target 2, and the gene-edited line KO2 with base substitution “G-C” at target 2 and was deleted with the “T” base at target 1 (Fig. S7). The results showed that *OAS-TL3* gene expression, OAS-TL enzyme activity, Cys content, and GSH content of *OAS-TL3* gene overexpressed soybean roots were increased at seedling stage. However, the transgenic lines with edited *OAS-TL3* gene were different (Fig. 3). The experimental results are consistent with Youssefian, Noji and Assylay (Youssefian et al. 1993; Noji et al. 2001). Available research suggests that OASTLs can act as a major L-cys desulfhydrase (Kurmanbayeva et al. 2022). Cys synthase is a key rate-limiting enzyme in the Cys synthesis pathway (Youssefian et al. 1993). It is also one of the precursors of GSH synthesis, which strongly regulates the synthesis of GSH (Strohm et al. 1995). Therefore, increasing *OAS-TL3* gene expression may increase Cys synthase activity and then increase Cys content. The increase of Cys content promotes GSH production.

GSH is indispensable in plant metabolism and has antioxidant and metabolic balance regulation (Foyer and Noctor 2011; Noctor et al. 2012). Analysis of GSH-deficient Arabidopsis mutant surface type showed that knockout of GSH2, which encodes GSH synthase, resulted in seedling death (Pasternak et al. 2008). The content of GSH in the *rwl* mutant was less than 5% of that in the wild type. It strongly inhibited root meristem development (Vemoux et al. 2000), and the lateral root density of the GSH synthetic mutant was lower than that of the wild type (Marquez-Garcia et al. 2014). In this study, the root data of transgenic lines showed that the total root length, projected area, surface area, total bifurcation number, and total crossover number were significantly higher in the overexpressed lines than in the control material WT, while the edited expressed lines showed the opposite (Fig. 4b). The reason is that GSH is essential for plant development and plays an important role in embryo and meristem development (Frottin et al. 2009; Bashandy et al. 2010). It is possible that the increased content of GSH in *OAS-TL3* transgenic soybean roots promotes root growth and morphological formation. To further verify the above conjecture, we externally applied different concentrations of reduced GSH to WT receptor materials, and the root data showed that GSH promoted the growth of soybean roots and morphological formation (Fig. 5b). Studies have also shown that exogenous addition of reduced GSH can induce adventitious root formation (Tyburski and Tretyn 2010) and lateral root formation (Zhu et al. 2016). These results further proved that root growth could be promoted by GSH.

The root system of land plants has two basic functions: the acquisition of necessary resources (e.g., water and nutrients) and resource delivery. The uptake of soil moisture and mineral nutrient mainly depends on the crop root system, and the morphological characteristics and spatial distribution of the root system play an important role in plant growth (Wang and Zhang 2015). We investigated the dry matter quality of the above-ground part of each transgenic line at the seedling stage. The results showed that the dry matter quality of the overexpressed line at the seedling stage was significantly higher than that of the control line, while the gene-edited line showed different dry matter quality (Fig. 4d). Studies have shown that higher root activity maintains a higher nutrient absorption capacity in the root system (Li et al. 2005). The root activity measurement results of all lines showed that the transgenic lines had significantly higher root activity than the control lines, while the gene-edited lines had different root activity (Fig. 4c). It further proves that the developed root system is beneficial to the absorption of nutrients in the soil and promotes the growth of plants.

The relative expression levels of the *OAS-TL3* gene in roots, stems, and leaves of all transgenic lines at flowering and bulking stages were measured in the experimental field, and it was found that the relative expression levels of the *OAS-TL3* gene were higher in the same parts during bulking stage. Furthermore, the expression levels in roots and leaves were higher than those in stems (Fig. 6). The protein content and amino acid composition of mature soybean seeds were determined. The results showed that the protein content of the overexpressed lines was significantly higher than that of the control material WT. In contrast, the gene-edited lines showed a significant increase in the content of the first limiting amino acid Met. The content of Cys was not significantly different (Table 1). As the first limiting amino acid, Met limits and reduces the availability of other amino acids. We hypothesized that increasing Met content in grain protein increases the total protein content.

**Fig. 7:**
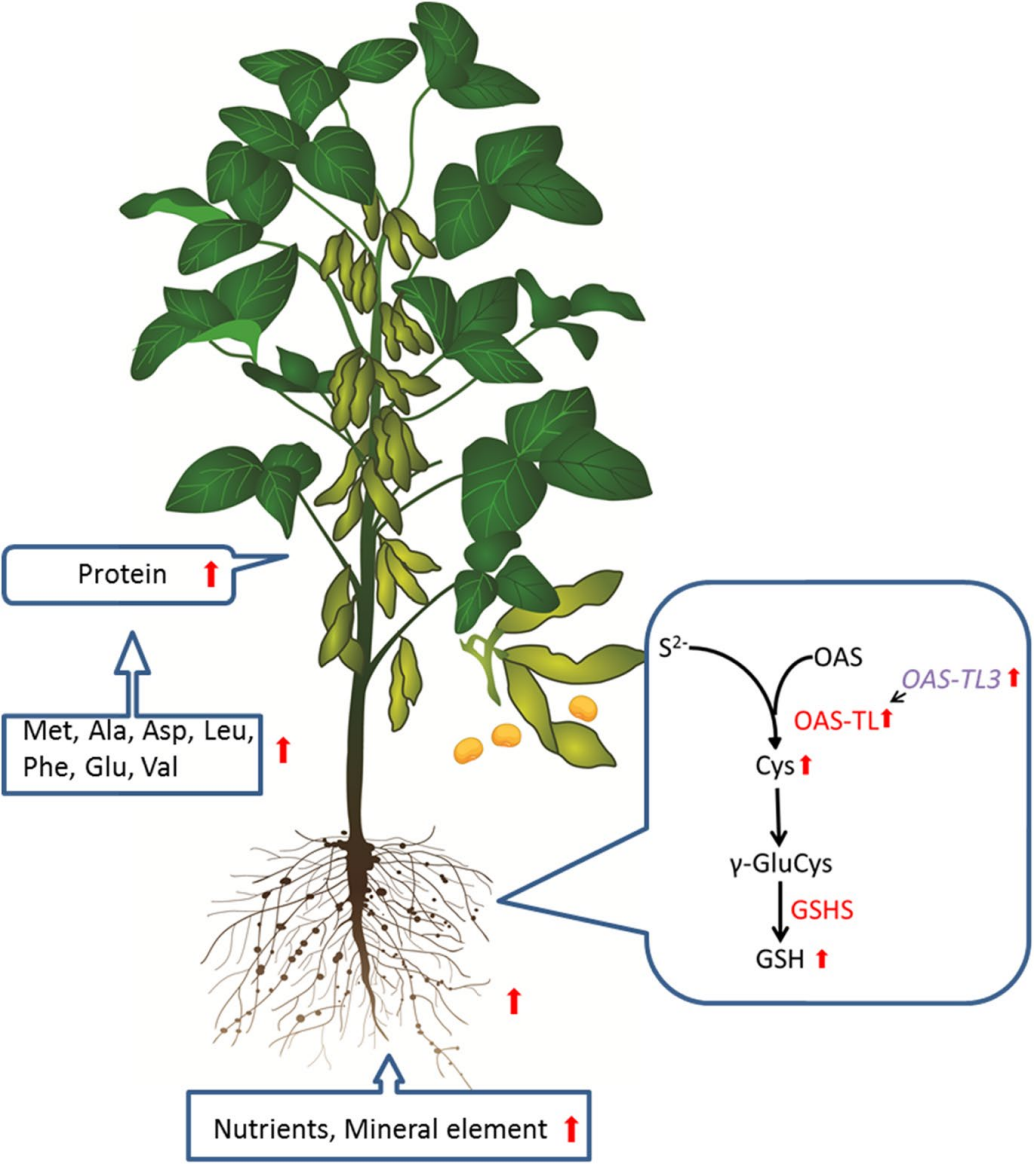
Soybean *OAS-TL3* gene regulates sulfur assimilation process, promotes root growth and development, and improves grain protein content schematic model. Enzymes are indicated in red characters. Elevations are indicated by red arrows. Abbreviations of metabolites: Cys, cysteine; γ-GluCys, γ-glutamylcysteine; GSH, glutathione

Based on the bidirectional verification of *OAS-TL3* gene overexpressed lines and gene-edited lines, we concluded that the expression of the *OAS-TL3* gene in soybean roots regulates the OAS-TL enzyme activity in the root system, which in turn regulates the production of Cys, one of the precursors of GSH. Indirect regulation and control of soybean root system growth have a promoting effect on GSH content. In order to regulate the growth and development of the soybean root system and the uptake of nutrients such as the trace minerals from the soil, the Met restrictive amino acid content needs to be firstly increased to break the restriction of Ala, Asp, Leu, Phe, Glu, and Val. As a result, the total protein content in grains can be increased (Fig. 7), and its mechanism needs to be further clarified in subsequent studies.

## Supplementary Information

ESM 1 (DOCX 13.6 kb)

ESM 2 (DOCX 1.10 kb)

## Data Availability

The materials presented in this article will be freely available to any researcher wishing to use them for non-commercial purposes, and the author responsible for distribution of materials in accordance with the policy described in the Instructions for Authors (www. springer. com) is Pi-wu Wang (peiwuw@163. com).
