# Supplementary material for: Enhancement of root sulfur metabolic pathway by overexpression of OAS-TL3 to increase total soybean seed protein content
Source: Mol Breed. 2023 Jan 12;43(1):4. doi: 10.1007/s11032-022-01348-y (PMC10248623; doi:10.1007/s11032-022-01348-y)
Supplement: Supplementary file 1 — (DOCX 13.6 kb) [file 11032_2022_1348_MOESM1_ESM.docx]

| Primer name | Primer sequence |
| --- | --- |
| 552Bar | F: 5′ TCAAATCTCGGTGACGGGC 3′ |
| 552Bar | R: 5′ ATGAGCCCAGAACGACGCC 3′ |
| 35sHm2 | F: 5′ GGGTTCTTATAGGGTTTCGC 3′ |
| 35sHm2 | R: 5′ CATATCCACGCCCTCCTACA 3′ |
| NOS | F: 5′ GAATCCTGTTGCCGGTCTTG 3′ |
| NOS | R: 5′ TTATCCTAGTTTGCGCGCTA 3′ |

Table.S1. Sequence list of PCR primers

The above primers were synthesized by Kumei Biotechnology Co., LTD., Jilin Province, China.
