# Supplementary material for: Enhancement of root sulfur metabolic pathway by overexpression of OAS-TL3 to increase total soybean seed protein content
Source: Mol Breed. 2023 Jan 12;43(1):4. doi: 10.1007/s11032-022-01348-y (PMC10248623; doi:10.1007/s11032-022-01348-y)
Supplement: Supplementary file 2 — (DOCX 1.10 kb) [file 11032_2022_1348_MOESM2_ESM.docx]

**Fig.S1** PCR detection of recombinant vector
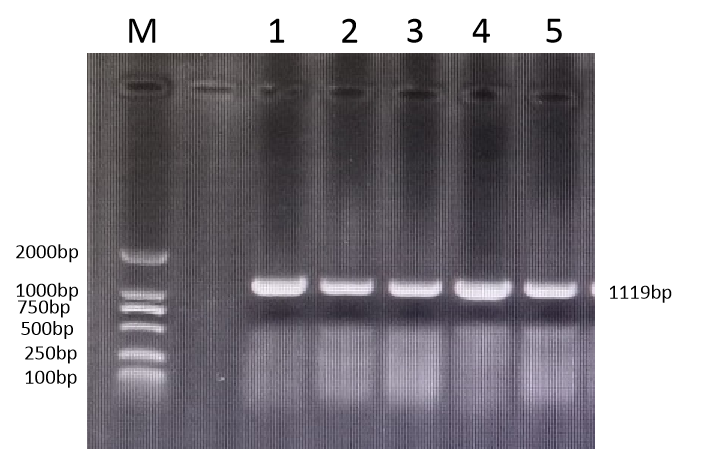
.(M:DNA Marker 1-5: Recombinant vector )

**
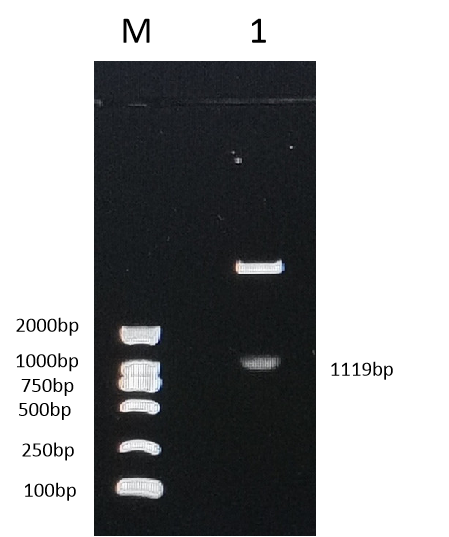
****Fig.S2** Validation of recombinant plasmid by enzyme digestion.(M:DNA Marker 1: Recombinant plasmid )


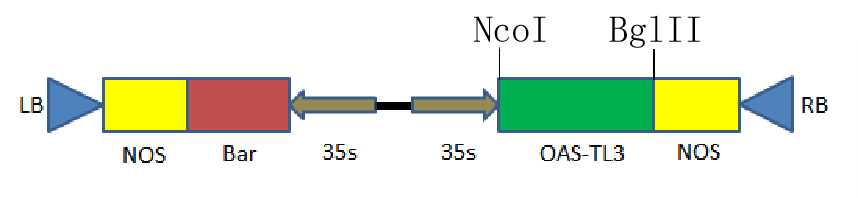
**Fig.S3** The schematic diagram of pCAMBIA3301-OAS-TL3 binary vector. (NOS terminator: NOS terminator is used for the termination signal of the gene expression, 35S: promoter used to express the glufosinate resistant gene, Bar gene: A reporter herbicide resistance gene, LB: left border and RB: right border)


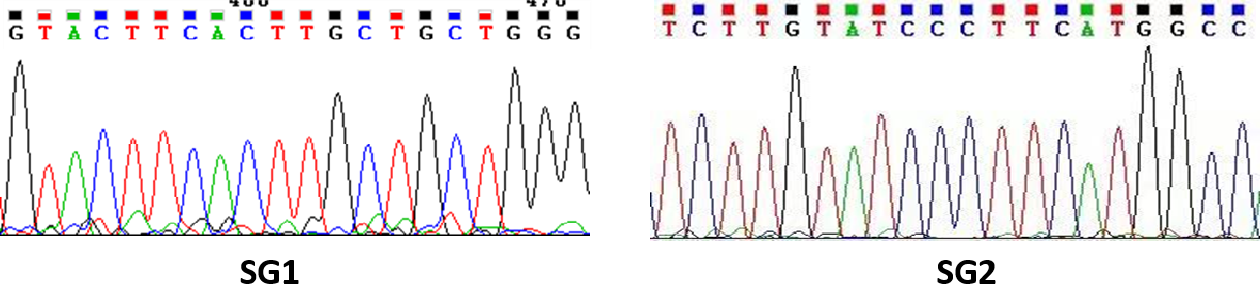
**Fig.S4** Edit target sequence alignment.


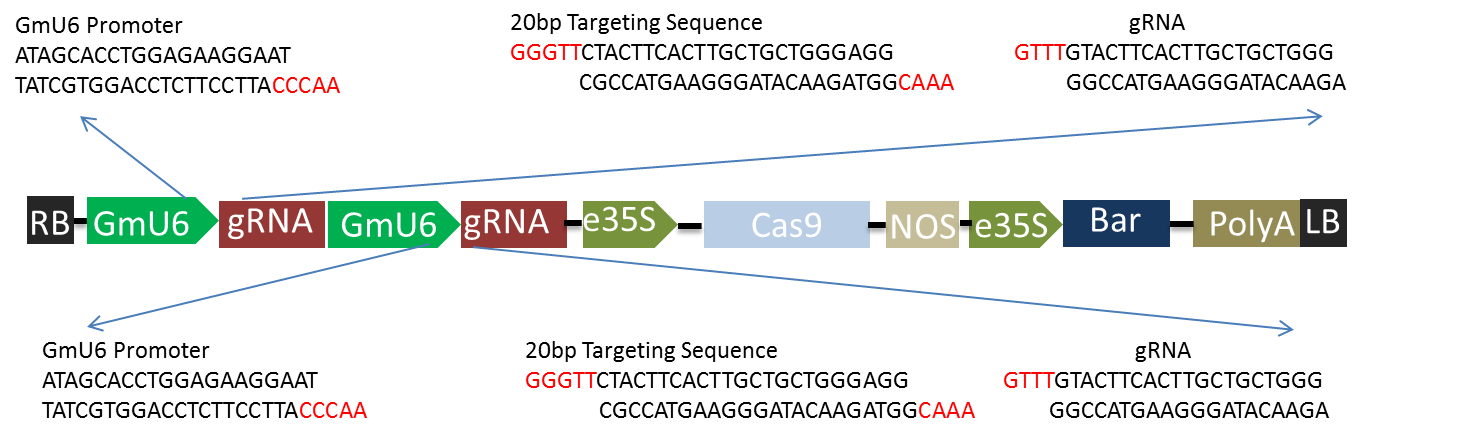


**Fig.S5** The schematic diagram of pCBSG015-OAS-TL3 binary vector. This system includes 20 bp long Synthetic guide RNA (sgRNA), NOS terminator: NOS terminator is used for the termination signal of the gene expression, small U6 expression cassettes derived from soybean, CaMV 35S: promoter used to express the glufosinate resistant gene, BAR gene: A reporter herbicide resistance gene, LB: left border and RB: right border


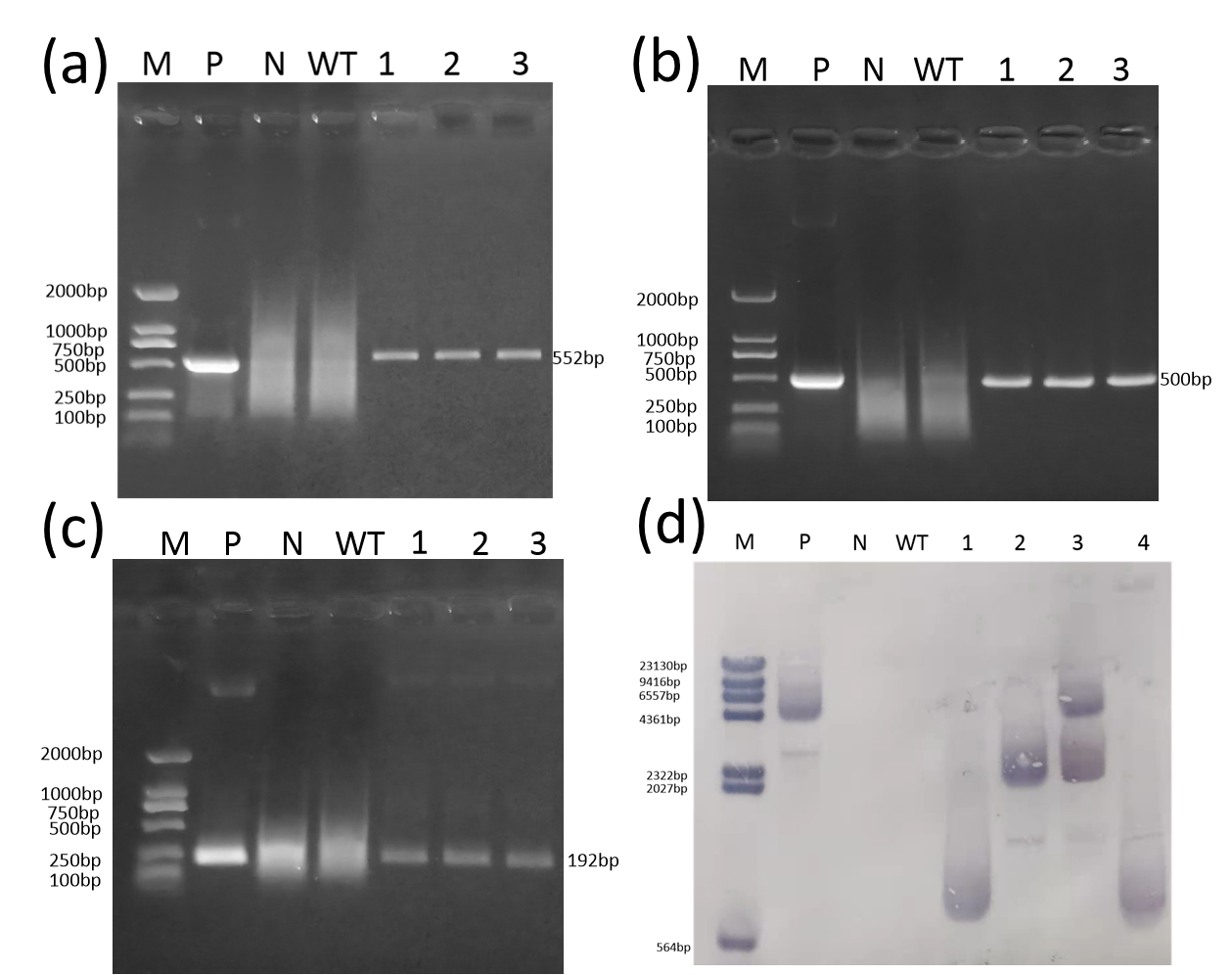


**Fig.S6** Molecular biological detection of overexpressed lines. **a** Bar gene test.(M:DNA Marker DL; P: plasmid; N:negative control; WT:contrast; 1-3: transgenic seedling.). **b** Promoter 35S detection. (M: DNA Marker DL; P: plasmid; N:negative control; WT:contrast; 1-3: transgenic seedling.). **c** NOS terminator detection (M: DNA Marker DL; P: plasmid; N:negative control; WT:contrast; 1-3: transgenic seedling.). **d** Southern blot hybridization of transgenic plants with BAR probe. The genomic DNA of 4 transgenic plants selected for detection followed by digestion of genomic DNA with restriction enzymes (HindIII and Tengo buffer). Where M represents marker, (P) positive control of plasmid, (W): Wild type DNA, and from 1 to 4 demonstrate different transgenic plants)

**Fig. S**
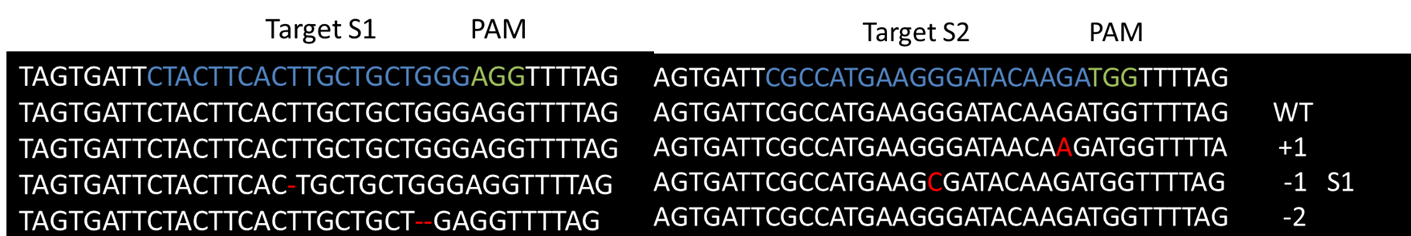
7 Sequences analysis revealed efficient targeted genome editing using CRISPR-Cas9 system. (Red letters in the sequence indicate mutation where, S: indicate substitution, +: represent addition or insertion while/−: indicate deletion.)
